# Molecular Mechanisms of Intestinal Adaptation in Short Bowel Syndrome: A Comprehensive Review

**DOI:** 10.3390/ijms27052105

**Published:** 2026-02-24

**Authors:** Dušan Radojević, Mihailo Bezmarević, Maja Pešić, Bojan Stojanović, Miloš Stanković, Mladen Pavlović, Nenad Marković, Marijana Stanojević-Pirković, Jelena Živković, Branko Anđelković, Ivan Radosavljević, Natalija Vuković, Nikola Mirković, Stefan Jakovljević, Mladen Maksić, Irfan Ćorović, Marina Jovanović, Nataša Zdravković, Danijela Jovanović

**Affiliations:** 1 Department of Internal Medicine, Faculty of Medical Sciences, University of Kragujevac, Svetozara Markovica 69, 34000 Kragujevac, Serbia; radojevicdusan@yahoo.com (D.R.);; 2 Clinic for Gastroenterology and Hepatology, University Clinical Centre Kragujevac, Zmaj Jovina 30, 34000 Kragujevac, Serbia; 3 Department of Hepatobiliary and Pancreatic Surgery, Clinic for General Surgery, Military Medical Academy, 11000 Belgrade, Serbia; bezmarevicm@gmail.com; 4 Faculty of Medicine of the Military Medical Academy, University of Defence, 11000 Belgrade, Serbia; 5 Center for Laboratory Diagnostics, University Clinical Centre Kragujevac, Zmaj Jovina 30, 34000 Kragujevac, Serbiamarijanas14@gmail.com (M.S.-P.); 6 Clinic for Surgery, University Clinical Centre Kragujevac, Zmaj Jovina 30, 34000 Kragujevac, Serbiaivanradoskapi@gmail.com (I.R.); stefan_jakov87@yahoo.com (S.J.); 7 Department of Surgery, Faculty of Medical Sciences, University of Kragujevac, Svetozara Markovica 69, 34000 Kragujevac, Serbia; 8 Department of Medical Biochemistry, Faculty of Medical Sciences, University of Kragujevac, 34000 Kragujevac, Serbia; 9 Clinic for Anesthesiology, Reanimation, and Intensive Care, University Clinical Center Nis, 18000 Niš, Serbia; 10 Center for Molecular Medicine and Stem Cell Research, Faculty of Medical Sciences, University of Kragujevac, 34000 Kragujevac, Serbia; 11 Department of Internal Medicine, General Hospital of Novi Pazar, 36300 Novi Pazar, Serbia; 12 Center for Anesthesiology, Reanimatology, and Intensive Care, University Clinical Center Kragujevac, Zmaj Jovina 30, 34000 Kragujevac, Serbia

**Keywords:** short bowel syndrome, GLP-2, intestinal adaptation, intestinal failure, malnutrition, VEGF, stem cell niche

## Abstract

Short bowel syndrome (SBS) develops when the remaining intestine is unable to sustain adequate nutrient and electrolyte absorption following extensive bowel resection. The condition is characterized by malabsorption and significant fluid losses which lead to dehydration and progressive weight loss, thus promoting patient dependence on parenteral fluids or nutrition. After an initial acute phase marked by accelerated intestinal transit and gastric hypersecretion, long-term clinical outcomes are largely determined by the capacity of the remaining bowel for intestinal adaptation—a sustained process of structural, functional, and molecular remodeling that enhances absorptive efficiency and restores fluid and nutrient homeostasis. This review summarizes the key histological and cellular features of the adaptive response, including crypt and villus remodeling, mucosal hyperplasia, and smooth muscle hypertrophy, and integrates emerging concepts in crypt biology that define the dynamic cross-talk between intestinal stem cells and the mesenchymal niche, together with their upstream regulatory pathways.

## 1. Introduction

Short bowel syndrome (SBS) is defined as a clinical condition resulting from extensive intestinal resection, typically characterized by a residual small bowel length of less than 200 cm with attendant malabsorptive symptoms, or by comparable clinical manifestations in patients with a longer remaining bowel length, a scenario commonly referred to as functional short bowel syndrome [1,2]. Characteristic clinical features of short bowel syndrome include high-output stoma losses, malnutrition with dependence on parenteral fluids or nutrition, progressive weight loss accompanied by micronutrient deficiencies, recurrent dehydration with electrolyte disturbances, and postprandial worsening of diarrhea due to the limited absorptive capacity of the remnant intestine in certain cases [3]. Short bowel syndrome carries a significant mortality burden, with reported 10-year survival rates ranging from 54.3% to 59% in patients with chronic intestinal failure [4,5]. Massive bowel resection triggers a complex cascade of pathophysiological responses in the remaining gastrointestinal tract. The immediate consequences include accelerated intestinal transit, gastric hypersecretion, and impaired fluid and electrolyte absorption, resulting in high-output fluid losses. These disturbances reflect the abrupt reduction in absorptive surface area and loss of ileal and colonic brake mechanisms, which together exacerbate malabsorption and dehydration in the early post-resection period [6,7]. This initial maladaptive state is followed by intestinal adaptation, a sustained process of structural, functional, and molecular remodeling of the remnant bowel that progressively increases absorptive efficiency and partially or fully restores fluid and nutrient homeostasis. One of the earliest documented observations of intestinal adaptation came from patients who underwent jejuno–ileal bypass surgery, a now obsolete bariatric procedure, in which a markedly shortened bowel was created intentionally, thus inducing malabsorption and weight loss [8]. Intestinal adaptation is a complex life-saving compensatory mechanism in patients with SBS. It is characterized by coordinated mucosal hypertrophy and hyperplasia, accompanied by remodeling and thickening of the muscularis propria, processes driven by integrated signaling between luminal stimuli, enteroendocrine L cells, the intestinal stem cell niche, and mesenchymal cell populations. This review provides a comprehensive, up-to-date synthesis of molecular mechanisms implicated in intestinal adaptation in SBS.

## 2. Histopathological and Ultrastructural Changes

The small intestine exhibits a proximal-to-distal gradient in healthy individuals. The jejunum possesses a markedly greater absorptive surface than the ileum, as both the plicae circularis and villi progressively diminish in prominence along the transition from jejunum to ileum. Ileum also has a physiologically inferior digestive and absorptive capacity, which may be in part due to lower availability of the nutrients in the ileum than in jejunum [9,10]. Studies following series of patients after massive bowel resection showed that the remaining functioning small bowel undergoes hypertrophy of the villi, hyperplasia of epithelial cells, and deepening of the intestinal glands. On electron microscopy, a dramatic hypertrophy of the microvilli was observed. Macroscopic alterations increased progressively over time, reaching their maximal extent at the two-year mark following the surgical procedure. Additionally, a decrease in bowel motility was observed after one year of surgery, with doubling or tripling of the caecum transit time in human studies [11,12,13,14]. Another study compared human colon histology between SBS patients and healthy controls and demonstrated a statistically significant increase in crypt depth (35 ± 20%) and total number of epithelial crypt cells (22 ± 16%). The proliferation rate of colonic cells was not different between healthy subjects and patients with SBS after 9 years [15]. Although not all studies reported macroscopic differences, their designs varied considerably [16]. Ziegler et al. showed no significant difference in crypt depth, villus height, and villus width in the small intestine between healthy subjects and patients with short bowel syndrome dependent on parenteral nutrition. However, despite matching for age and sex, biopsies were not obtained from anatomically corresponding regions, resulting in comparisons between the distal duodenum of controls and the jejunum or ileum of patients with SBS. In addition, multiple other factors—including diet, age, comorbidities, medications, and environmental influences—may contribute to interindividual variability in intestinal morphology [17]. Animal models provide more robust evidence of colonic mucosal hyperplasia in the SBS [18,19,20]. Complementary to histologic assessment, measurement of plasma citrulline levels provides a quantitative indicator of intestinal adaptation. Citrulline is a product of enterocyte metabolism and a surrogate marker of enterocyte mass [21]. Since plasma citrulline levels strongly correlate with the remaining small bowel length, plasma citrulline may be used to assess risk of lifelong dependence on parenteral nutrition [22,23]. Similarly, Apo-protein IVA (APO IVA) is a protein synthesized exclusively by enterocytes in the ileum which may be also measured in the serum of SBS patients and serve as an additional marker of enterocyte mass [24].

Structural adaptation of the remnant intestine extends well beyond the mucosal compartment. Khasanov et al. demonstrated that the muscularis propria undergoes marked thickening in both the jejunum and ileum, driven predominantly by smooth muscle hypertrophy and accompanied by increased collagen deposition. Although the mechanisms underlying this hypertrophic response remain incompletely defined, the same study reported a twofold increase in nestin-expressing cells within the myenteric (Auerbach’s) plexus—cells regarded as neural progenitors—suggesting that enhanced enteric neuroplasticity may contribute to post-resection intestinal remodeling [25].

## 3. Intestinal Crypts: Epithelium and the Mesenchyme

Both human and animal studies demonstrate that the extent of resection correlates closely with the degree of intestinal adaptation [26,27,28]. The presence of the distal ileum and colon further determines the adaptive response in patients with SBS [29]. The divergence in intestinal adaptive capacity is largely attributable to the regional distribution of enteroendocrine L-cells in the bowel, which represent a key driver of intestinal adaptation. L-cells are most densely located in the ascending and transverse colon, followed by the terminal ileum, whereas they are relatively sparse in the duodenum and jejunum [30]. Consequently, the adaptive potential of patients without functional distal bowel is markedly attenuated. Clinical studies of human SBS patients without terminal ileum, the ileocecal valve and/or colon demonstrate impaired intestinal adaptive response that is reflected in significantly higher rates of PN dependence [31,32]. As central mediators of intestinal adaptation, L-cell–derived hormones are strongly influenced by luminal nutrient exposure. Small bowel crypts are populated by stem cells and Paneth cells [33]. Following proliferation in the crypts, progenitor transit amplifying (TA) cells migrate away through the transit-amplifying zone, undergo definitive differentiation to enterocytes, and complete their life cycle in the next 3 to 7 days in the intestinal villi. Intestinal stem cells (ISCs) that express Leucine-rich repeat-containing G protein-coupled receptor 5 (Lgr5^+^) constitute a rapidly-cycling population responsible for continuous epithelial renewal, whereas more quiescent Lgr5^low^ cells, often referred to as +4 cells, are thought to contribute to epithelial regeneration in response to injury. Paneth cells provide signals through Wnt3, R-spondin (RSPO), and epidermal growth factor (EGF), secreted ligands that bind to their receptors (Frizzled and LRP5/6, Lgr5, and EGFR) located on the surface of Lgr5^+^ ISCs. These signals are crucial for the maintenance of Lgr5^+^ stem cells [34,35]. Beyond the epithelial compartment, the surrounding mesenchyme forms a critical component of the crypt niche. The mesenchyme, primarily located in the lamina propria immediately adjacent to crypt and villus epithelium, is being increasingly studied for its roles in regulating crypt cell proliferation and differentiation. It comprises the extracellular matrix and diverse cell populations, including Foxl1^+^ telocytes, GLI1^+^ cells, PDGFRα^+^ mesenchymal cells, intestinal subepithelial myofibroblasts (ISEMFs), and fibroblasts. Together, these populations coordinate epithelial renewal by regulating the balance between proliferative and differentiative signaling.

ISC proliferation is driven by Wnt/β-catenin signaling, whose activity is locally reinforced by R-spondins produced by a specialized CD81^+^ PDGFRα^lo^ fibroblast population known as trophocytes [36]. While Paneth cells represent the principal source of Wnt ligands in the small intestine, this function is assumed by GLI1^+^ mesenchymal cells in the colon, which also act as a reserve source of Wnt signaling in the small intestine [37]. On the other hand, APC is a negative regulator of β-catenin, and alongside other inhibitory signals such as BMPs, contributes to a reduction in proliferation as cells migrate up the crypt-villus axis. Hedgehog (Hh) signaling from differentiated epithelial cells further modulates mesenchymal cell behavior, coordinating the epithelial–mesenchymal cross-talk that establishes the proliferation and differentiation gradient. This spatial signaling gradient—characterized by high Wnt and low BMP activity at the crypt base and progressively increasing BMP signaling toward the villus—maintains the balance between proliferation and differentiation [38]. Low BMP activity at the crypt base is maintained through both reduced BMP production and local expression of BMP antagonists such as Noggin and Gremlin, thereby permitting stem cell expansion. In contrast, increasing Hedgehog activity toward the villus promotes BMP signaling via PDGFRα^hi^ telocytes, driving terminal differentiation and limiting proliferation. In the colon, Hedgehog signaling antagonizes Wnt activity, further reinforcing regional differences in epithelial renewal dynamics [36,39].

## 4. Stem Cell Niche Changes

The stem cell niche in the intestinal crypts undergoes profound remodeling following intestinal resection and development of the short bowel syndrome. Gazit et al. have examined the later phases of intestinal adaptation in humans (from 6 months to 10 years post resection). They demonstrated that the stem cell niche in patients with short bowel syndrome is characterized by increased crypt cellularity, driven predominantly by expansion of the Lgr5^+^ stem cell population, without a concomitant increase in the +4 reserve stem cell compartment. This suggests that +4 stem cells do not play a role in intestinal adaptation, at least in the chronic phase. Several gut mesenchymal cell subtypes contribute to the stem cell niche. These include ISEMFs that express epimorphin, a regulator of growth factor secretion; FOXL1^+^ telocytes, known to support stem cell function in both mouse models and human IBD. Additional populations described in mouse intestine include a heterogenous group of PDGFRα^+^ cells. Despite their heterogeneity, these cell groups share PDGFRα expression and collectively serve as the principal mesenchymal sources of non-canonical Wnt ligands (Wnt2b, Wnt4, Wnt5a), R-spondins (R-spondin1, R-spondin2, R-spondin3), and BMPs, which together coordinate epithelial growth and regeneration. In patients with SBS, stromal ISEMFs respond locally to the resection by inhibiting BMP4 secretion, therefore allowing for increased crypt cell proliferation. Although increased Wnt ligand expression was observed, Wnt signaling was not increased in patients with SBS, which could be explained by a concomitant increase in BMP signaling [40,41,42,43]. Following intestinal resection, expression of Hh is down-regulated. Consistent with its previously discussed role, experimental inhibition of the Hh pathway resulted in increased enterocyte migration speed, and apoptosis, but had no effect on proliferation and crypt and villus morphology. These findings suggest that while Hedgehog suppression accompanies intestinal adaptation, it is not an essential driver of the adaptive process [44]. L-cell progenitor proliferation also appears to be influenced by the local presence of GLP-1 [45]. Importantly, these alterations extend beyond the intestinal stem cell compartment. In murine models of SBS, increased vitamin A exposure drives a transcriptional reprogramming of differentiated distal enterocytes toward a proximal intestinal phenotype, marked by increased expression of *Creb3l3* and *Klf4*, a process referred to as ‘proximalization’ [46].

Recently, the development of intestinal organoid models has enabled detailed investigation of the complex cellular and molecular interactions within the intestinal crypt niche. Organoids are three-dimensional, self-organizing cellular systems derived from stem or progenitor cells that recapitulate key structural, molecular, and functional features of their tissue of origin. By preserving intrinsic programs of epithelial self-renewal and lineage specification, organoids provide an intermediate model between conventional two-dimensional cultures and in vivo systems. Among these, intestinal organoids faithfully reproduce essential aspects of crypt–villus organization and epithelial differentiation. They are generated from isolated Lgr5^+^ intestinal stem cells or tissue-derived crypts that are embedded in a three-dimensional extracellular matrix scaffold and cultured under defined growth factor conditions that support stem cell maintenance and lineage specification [47]. Organoids are typically maintained within an extracellular matrix surrogate such as Matrigel, a laminin-rich basement membrane extract containing collagen IV, entactin, and heparan sulfate proteoglycans, which provides mechanical support and permissive biochemical cues. Owing to the absence of native mesenchymal, immune, neuronal, and vascular compartments, intestinal organoids require exogenous supplementation with key niche-derived factors—including Wnt3a, R-spondin-1, epidermal growth factor, and the BMP antagonist Noggin—to maintain stem cell identity and sustain epithelial self-organization [47]. Organoids have emerged as a powerful platform for dissecting epithelial-intrinsic mechanisms of intestinal development, regeneration, and disease. Gazit et al. demonstrated that intestinal stem cells isolated from SBS patients retain an enhanced enteroid-forming capacity after ex vivo culture and re-plating, indicating a persistent, cell-intrinsic alteration in stem cell behavior that is maintained outside the native luminal and mesenchymal environment and is consistent with durable epigenetic remodeling [40]. In a series of studies led by Sato and Sugimoto, intestinal organoid transplantation was explored as a regenerative strategy for SBS. In their initial work, ileal organoids were shown to engraft ectopically and confer ileal characteristics to non-ileal intestinal segments, providing proof of principle that regional epithelial identity can be transferred by organoid-based approaches. Building on this concept, their subsequent study demonstrated that efficient engraftment requires preservation of the native stromal niche. By selectively removing the jejunal epithelium while maintaining the underlying mesenchymal compartment, ileal organoids could efficaciously engraft within the jejunum, resulting in functional “ilealization” of the recipient segment. This niche-preserving strategy restored bile acid absorption, improved lipid handling, and led to a marked survival benefit in a rat model of SBS, thereby establishing organoid-based epithelial replacement as a potential regenerative approach for intestinal failure [48,49]. Although recent advances have enabled the generation of intestinal organoids comprising derivatives of all three germ layers and demonstrating peristaltic activity, these systems have not yet been evaluated in the context of SBS. Further investigation may provide a foundation for the development of translational regenerative strategies for intestinal failure [50]. The epigenetic alterations in SBS have only been partially characterized; however, changes in microRNA (miR) expression have been observed during the acute post-resection phase. Specifically, miR-125a and miR-214 are upregulated within the intestinal crypts by approximately 2.4-fold and 3.2-fold, respectively. miR-125a promotes apoptosis through translational suppression of the prosurvival protein Mcl-1. Although apoptosis is often considered deleterious, a transient increase in programmed cell death may contribute to tissue remodeling during the early stages of intestinal adaptation. The role of miR-214 in intestinal adaptation, however, has not yet been elucidated. [51]

## 5. Angiogenesis in Intestinal Adaptation

Angiogenesis is a critical part of the intestinal adaptation. Adapted villi and submucosal compartments show evidence of neovascularization after intestinal resection [52]. Immediately following intestinal resection, increased oxygen uptake is observed alongside a decrease in intestinal blood flow [53]. Since hypoxia is known to be a strong proangiogenic stimulus, increase in hypoxia inducible factors (HIF) was expected and observed [54]. Rowland et al. demonstrated an acute increase in HIF 1 alpha immediately following massive intestinal resection, but not its downstream target, vascular endothelial growth factor (VEGF), one of the key mediators of angiogenesis. As previously mentioned, saliva provides crucial EGF and VEGF to the intestine following massive bowel resection. Deprivation of VEGF blunts both the angiogenic and adaptive response of the intestine, suggesting a crucial role of VEGF in SBS patients [55]. C-X-C motif chemokine ligand 5 (CXCL5) is a proangiogenic chemokine that is expressed at increased levels in the lamina propria following massive intestinal resection [56]. Although CXCL5 is a crucial angiogenic factor in SBS, it is not crucial in intestinal epithelial proliferative response [57]. However, angiogenesis appears to be a supportive response to the increased metabolic needs of the adapting intestine, since Diaz-Miron et al. demonstrated that CXCL5 disrupts angiogenesis but not structural changes of the bowel [58]. Neovascularization is still necessary for complete adaptation; CXCL5 knock-out mice with hindered angiogenesis had downregulated PepT1 expression, impaired fat absorption and slower postoperative weight gain, compared to controls [58].

## 6. Changes in Intestinal Nutrient Transporter Expression

Aside from gross macroscopic changes, important adaptive mechanisms occur in nutrient transporter expression. PepT1, an oligopeptide transporter, is normally expressed in the duodenum, jejunum, ileum and, to a lesser extent, the colon of healthy humans. PepT1 expression is inducible and some of the known upregulating factors are inflammation, acute malnutrition, refeeding, and high fat or high protein diets, while salinity and diurnal rhythm may also modulate its expression [59,60].

A study by Ziegler et al. clearly demonstrates the upregulation of PepT1 in the colonic mucosa of patients with SBS (a five-fold increase in PepT1 mRNA), suggesting a molecular basis of intestinal adaptation in the setting of intestinal failure [16]. In contrast, another study did not demonstrate a statistically significant increase in colonic PepT1 expression in patients with short bowel syndrome and instead attributed intestinal adaptation predominantly to mucosal hyperplasia and bowel dilatation [15].

These conflicting results demand a deeper dive into different study designs. The former study examined more acute effects of intestinal resection (only two out of seven patients had been on PN longer than 18 months), while the latter study examined 12 patients with a mean of 9.0 ± 5.6 years after restoration of bowel continuity, and only nine of the twelve patients had remained dependent on parenteral nutrition. The small number of patients, substantial variability in residual bowel length, and heterogeneity in the underlying etiology of short bowel syndrome across studies likely explains the conflicting results. Taken together, these factors do not allow for a definitive conclusion regarding PepT1 expression in the colon of patients with SBS. However, both human studies clearly demonstrated no significant increase in the small bowel PepT1 expression in SBS.

Experimental data from rodent models do not demonstrate a robust increase in colonic PepT1 expression comparable to that reported in human studies. However, direct extrapolation is limited by important anatomical differences, as rats possess a large cecum that may obscure colonic adaptive responses following intestinal resection [61]. In addition, PepT1 regulation may occur at the level of cellular trafficking rather than total protein abundance, with evidence suggesting translocation of PepT1 from intracellular pools to the apical membrane without a corresponding increase in overall expression [62,63,64]. Studies investigating PepT1 expression in the small intestine following massive resection further indicate that adaptive increases in peptide absorption are primarily driven by mucosal hyperplasia rather than transcriptional upregulation of PepT1 itself [61,65]. Although PepT1 expression is upregulated during states of mechanoluminal deprivation—an effect that is reversed by GLP-2, which acts as a physiological negative regulator of transporter expression [66,67]—available evidence suggests that PepT1 plays a limited role during the chronic adaptive phase of short bowel syndrome. This does not preclude a larger role for PepT1 during the early, post-resection phase, which remains insufficiently characterized. Similar results have been demonstrated in the studies of intestinal absorption of glucose via SGLT1 and GLUT2 transporters whose total expression was also found to be primarily increased through mucosal hyperplasia [62,68]. Brush border enzymes like TREH and MGAM are upregulated only in small bowel segments that are deprived of the mechanoluminal signaling [67]. In contrast, an experimental animal study of the acute phase of intestinal adaptation shows that SGLT1 expression in the ileum begins to rise within hours after a massive intestinal resection, and reaches maximum values at one week after surgery. This early response highlights the epigenetic influence on mature enterocytes and SGLT1 expression [69]. Current data from experimental models and human studies suggest that while both the small bowel and colon are capable of upregulating epithelial transporters, the chronic phase of intestinal adaptation is driven predominantly by mucosal hyperplasia and increased absorptive surface area rather than by enhanced transporter expression.

## 7. Determinants of Intestinal Adaptation

**Figure 1:**
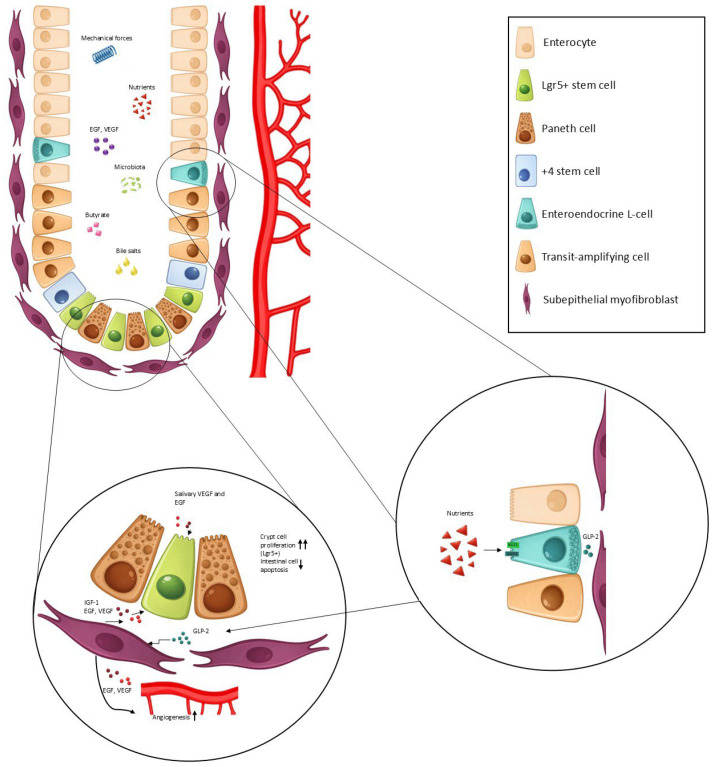
Key molecular drivers of intestinal adaptation. Intestinal adaptation is driven by a combination of luminal and mechanical cues, including nutrient exposure, bile acids, microbial-derived metabolites (notably short-chain fatty acids such as butyrate), and mechanical forces generated by luminal flow and stretch. Luminal nutrients activate specific transporters on enteroendocrine L cells, including SGLT1 and GLUT2, leading to the secretion of GLP-2. GLP-2 acts in a paracrine manner on subepithelial myofibroblasts within the lamina propria, inducing the release of trophic mediators such as IGF-1, EGF, and VEGF. In addition to local production, EGF and VEGF are also supplied exogenously via salivary secretions entering the gastrointestinal lumen. These growth factors promote angiogenesis, enhance mucosal perfusion, stimulate Lgr5^+^ intestinal stem cell proliferation, and suppress epithelial apoptosis, collectively driving structural and functional intestinal adaptation. EGF—epidermal growth factor, VEGF—vascular endothelial growth factor, IGF-1—insulin like growth factor 1, GLP-2—Glucagon-like peptide 2, ↑—upregulation, ↓—downregulation.

A shortened bowel is subject to many mechanical and biochemical stimuli. Mechanical forces can cause stretching of the bowel, which is a potent stimulus for adaptation. Additionally, the presence of nutrients modulates intestinal adaptation to a large degree. These signals are finally converted into paracrine stimuli which lead to molecular and histological changes observed in intestinal adaptation (Figure 1).

Mechanical drivers that contribute to intestinal adaptation are stretching forces. While there is limited human data on the exact impact of stretching forces in short bowel syndrome [70,71,72], mechanical luminal forces are known to induce intestinal adaptation in the remaining bowel. Many experimental biomechanistic studies have shown that mechanical distraction or luminal distension are potent stimuli for promoting intestinal adaptation in the remaining bowel [73,74]. Peristalsis and shear stress also modulate mechanosignaling [75]. Distractional enterogenesis is a field that is being increasingly studied in preclinical studies in the treatment of short bowel syndrome [76]. In an experimental study on a defunctionalized intestinal segment in mice, spring insertion increased crypt depth and villus height, while displaying increased expression of MYH11 and OLFM4, indicating smooth muscle hypertrophy alongside increased crypt cell density and proliferation [76].

### 7.1. Nutrient Uptake

By far, the most important extrinsic driver of intestinal adaptation is oral nutrient uptake. In general, intestinal mucosa depends on intraluminal nutrients, and experimental studies consistently demonstrate mucosal atrophy in fasting states which is disproportionate to weight loss during that period. In the absence of mechanoluminal stimulation, LGR5^+^, OLFM4^+^, and SOX9^+^ intestinal stem cells decrease in activity and proliferation, despite adequate systemic energy supply [67,77]. Additionally, fasting disturbs epithelial ion transport and increases fluid and electrolyte losses through the intestine [10,78,79,80,81,82]. Although total parenteral nutrition causes upregulation of peptide transporter molecules, enterocyte loss and mucosal atrophy overwhelm this effect and the net effect is diminished nutrient uptake [10,66]. Numerous studies have demonstrated superior intestinal adaptation in patients receiving enteral nutrition, compared to parenteral. Enteral feeding is, therefore, a fundamental requirement for intestinal adaptation [83]. Standard nutritional guidance for SBS patients emphasizes a hypercaloric oral diet incorporating all major macronutrient classes. However, not all macronutrients exhibit equal enteroplastic effects. High lipid intake is crucial to intestinal adaptation. Specifically, long-chain fatty acids are more effective in inducing enterocyte hyperplasia than medium chain fatty acids [84]. Saturated fatty acid intake after distal small-bowel resection led to a twofold increase in glucose uptake compared with polyunsaturated fatty acids in an experimental animal model. Saturated fatty acids are also a potent stimulus for villus height and crypt depth increase. Specifically, the addition of palmitic acid to the diet lead to an increase villus height and crypt depth in an experimental model [85,86]. Short chain fatty acids (SCFAs) also exhibit positive effects on the intestinal mucosa [87,88]. Butyrate, a short-chain fatty acid, has a positive effect on the physiology of gut epithelial barrier. In addition to exerting anti-inflammatory and immuno-modulatory properties, butyrate increases intestinal barrier integrity through suppressing the leaky Claudin-2 expression [89], and by increasing synaptopodin production, a molecule crucial for regulating intestinal permeability and mucosal wound healing [90]. Butyrate increases enterocyte proliferation and inhibits apoptosis in experimental animal models following intestinal resection, at least in part through increased GLP-2 secretion [91,92]. Addition of butyrate to parenteral nutrition solutions has dramatically reduced the associated intestinal atrophy with TPN in experimental animal models [87,88]. This effect is probably mediated by L-cell derived factors, since SCFAs are a potent stimulus of L-cell activity. Microbial derived SCFAs increase L-cell secretion of GLP-1 and PYY [93]. Butyrate has also been shown to increase intestinal smooth-muscle (ISM) mass in vivo and to enhance ISM proliferation in vitro through upregulation and activation of the YAP pathway [94,95]. Since SBS is accompanied by increased epithelial permeability [96], butyrate intake may be beneficial through more than one mechanism. Carbohydrates induce an adaptive enterocyte response that consists of increased crypt enterocyte turnover and migration rate, and increased density of glucose transporters. The increase in glucose transporters like GLUT5 and SGLT-1 starts in the crypt cells, which then migrate over the next 3 days up the villus, allowing for increased carbohydrate absorption [97]. Similarly, high protein diets increase the jejunum’s capacity to absorb amino acids [98]. Distinct adaptive patterns are observed in essential versus non-essential amino acid transport. High-protein diets preferentially enhance the absorptive capacity for non-essential amino acids, whereas essential amino acid transport increases to a lesser extent. Conversely, protein-deficient diets may upregulate essential amino acid uptake while concurrently reducing the transport capacity for non-essential amino acids in the small intestine [99]. Protein-deficient diet decreases villi height and intestinal permeability in healthy rats, which are restored when supplemented with glutamate. Dietary glutamate (GLM) is mostly metabolized by the intestine during the first pass [100], and has been shown to induce proliferation of the intestinal epithelium, in an GLP-2 independent fashion. Addition of GLM to the diet led to a dose-dependent decrease in circulating GLP-2 levels, potentially mediated either through post-transcriptional or post-translational regulation of GLP-2 secretion, suggesting a feedback interaction between the two mechanisms [101]. Parenteral glutamine is the primary energy source for the enterocytes, and the addition of glutamine to TPN has also reduced the intestinal atrophy associated with TPN [102]. An intestinal stem cell nutrient-sensing mechanism may also contribute to increased crypt cell proliferation through a pathway that couples nutrient availability to protein glycosylation and a metabolic shift toward glycolysis, thereby supporting stem cell proliferation, as demonstrated in a 2018 study on Drosophila intestine. This HBP-mediated metabolic rewiring modulates the responsiveness of ISCs to insulin receptor (InR)–TOR signaling, which leads to ISC proliferation [103]. Zinc deficiency is known to decrease mucosal renewal and increase mucosal permeability. A study demonstrated upregulation of a zinc transport gene, *SLC39A5*, in humans with SBS. In addition, in vitro, zinc increased ISC proliferation [104,105].

### 7.2. Bile Salts

In addition to exogenous nutrients, luminal bile salts may contribute significantly to the intestinotrophic effect. Ileal resection leads to a reduction in enterohepatic circulation of bile acids, and subsequently, a deficiency of the bile acid pool. This may lead to steatorrhea, which resolves upon oral bile acid intake [106,107]. Apart from their traditional effects and capacity to increase fat absorption, bile salts are receiving growing attention for their proposed role in short bowel syndrome. Small bowel and colon lose important stem cell proliferation stimuli in states with low bile salt influx, including fasting or TPN. Taurodeoxycholate, a water-soluble, deoxycholate taurine conjugate salt, induces crypt cell proliferation, and exerts an anti-apoptotic effect on the enterocytes through the activation of NF-kB [108,109,110].

### 7.3. Pancreatic Juice and Digestive Enzymes

Although early studies hypothesized the importance of pancreatic secretions in intestinal adaptation in the short bowel syndrome [111], extensive data on the importance of pancreatic juice in patients with short bowel syndrome is lacking. Some of the observed positive effects of pancreato-biliary secretions are now attributable to bile salts and not pancreatic enzymes. However, intake of commercially available enzyme products has been associated with a recovery of mucosal atrophy, and an increase in PepT1 expression in an animal model of exocrine pancreatic insufficiency [112]. Amylase specifically is believed to have a central role in the preservation of intestinal morphology. To our knowledge, the only interventional human study addressing this question included six paediatric and five adult patients with short bowel syndrome and found no significant overall improvement in enteral fat or protein absorption. Notably, several individual patients demonstrated improved absorption, suggesting that pancreatic enzyme supplementation may confer benefit in selected SBS patients [113]. Limited evidence in this field underscores the need for further research to clarify the role of pancreatic enzymes in short bowel syndrome. While pancreatic enzyme replacement therapy does not appear to play a central role in intestinal adaptation, it may be beneficial in selected clinical scenarios.

### 7.4. Bowel Microbiota

Gut microbiota is now known to play a role in many digestive and non-digestive diseases [114], and its role in patients with SBS has, therefore, become an important focus of current research. Unfortunately, the role and influence of microbiota in intestinal adaptation have not yet been untwined. So far, numerous studies have linked certain bacterial species with either better outcomes (high abundance of SCFA producing *Clostridium* cluster XIVa are linked to significantly shorter PN duration) [115,116], or worse outcomes (*Enterobacteriaceae* is associated with longer PN duration, while *Lactobacillus* and *Prevotella* are linked to high stomal output of >1500 mL per day) [117]. *Prevotella* has been associated with reduced intestinal concentration of a short chain fatty acid, acetate, and decreased intestinal IL-18 production thereby exacerbating intestinal inflammation. [118]. Notably, an interesting study demonstrated near-absent levels of butyrate in paediatric patients with SBS, alongside a reduction in commensal anaerobes known to produce SCFAs, like *Ruminococcaceae* and *Lachnospiraceae* [119]. *Bacteroidetes*, particularly the genus *Bacteroides*, are SCFA producing bacteria that are consistently reduced in patients with short bowel syndrome; however, when present, their abundance is associated with shorter dependence on parenteral nutrition [120,121]. One study found that CRP levels >3 mg/dL were inversely associated with *Bifidobacterium* presence in SBS patients, suggesting a possible protective role. *Bifidobacterium* has been shown to increase mucosal bowel integrity by up-regulating occludin, claudin-3, and ZO-1 expression [117,122]. Although a direct mechanistic link has not yet been fully established, mutations in the pattern-recognition receptor nucleotide-binding oligomerization domain–containing protein 2 (NOD2), a key mediator of bacterial sensing, are more frequently observed in patients with intestinal failure than in healthy individuals [123].

A recent systematic review of the available literature in paediatric short bowel syndrome concluded that SBS patients with either intestinal autonomy and PN dependence had significantly lower bacterial diversity and richness compared to healthy controls. Further research in this area is hindered by substantial methodological difficulties and study heterogeneity across sample collection, storage, and processing [124]. The definitive role of the gut microbiota in short bowel syndrome has yet to be established, as current studies do not demonstrate a causal link between microbial composition and intestinal adaptation.

### 7.5. Hormone Regulation

Hormonal regulation of intestinal adaptation represents one of the more studied areas in short bowel syndrome and intestinal failure and has led to the development of therapeutic agents which have revolutionized this clinical field (Table 1) [125]. Growth hormone (GH) was one of the first examined hormones in short bowel syndrome. It appears that GH contributes to the intestinal adaptation since experimental animal studies demonstrated a decreased adaptive response after bowel resection in mice that have undergone pituitary gland removal [126]. Administration of GH increased the small bowel absorptive capacity and mucosal hypertrophy when given alongside glutamate in rats with short bowel syndrome [127,128]. A study done on PN dependent SBS patients demonstrated increased absorption of all macromolecules when given low doses of GH [129]. Further studies, including a double-blind, randomized, placebo-controlled clinical trial, have shown positive effects of the combination of glutamine administration alongside growth hormone therapy in short bowel patients and allowed for a greater reduction in PN dependence in these patients [130,131,132]. In the early 2000s, this all has led to the FDA approval of somatropin, a recombinant human growth hormone, for treatment of short bowel syndrome in patients receiving specialized nutritional support [133]. Apart from GH, another hormone that is implicated in short bowel syndrome is aldosterone. Increased fluid and electrolyte loss from the gastrointestinal tract leads to reduced circulating volume, which then leads to renal hypoperfusion. Finally, a compensatory activation of the renin-angiotensin-aldosterone system occurs, leading to secondary hyperaldosteronism as a systemic adaptive response to hypovolemia [72,134].

Human epidermal growth factor (EGF) is a polypeptide with known positive effects on epithelial proliferation and differentiation. EGF also plays an important role in intestinal development, and is one of the most abundant growth factors present in the human milk [135,136]. EGF receptors (EGFR) are located on the apical and basolateral cellular membrane of the enterocytes. A physiologic increase in both EGF and EGFR after small bowel resection has been observed, with redistribution of EGFR from the basolateral membrane to the brush border membrane of the enterocytes [137]. EGF increases RNA, DNA, and protein synthesis, and also stimulates stem cell mitosis in the intestinal crypt. In general, EGF reduces gut permeability and increases villous height and crypt depth. EGF promotes mucosal proliferation by suppressing enterocyte apoptosis, a process thought to involve upregulation of anti-apoptotic bcl-w and downregulation of pro-apoptotic bax and p38 mitogen-activated protein kinase expression [138,139,140]. GLP-2 also induces the expression of other EGFR ligands like amphiregulin and epiregulin, which then activate EGFR on the enterocytes and lead to consequent engagement of downstream mitogenic and pro-survival signaling pathways [141]. A series of experimental studies demonstrated that neither EGFR nor IGF1R are obligatory for increasing crypt depth or villous height in intestinal adaptation [142]. It appears that EGF exhibits the strongest effects immediately after resection, and that EGF administration later on has a limited effect on intestinal adaptation [137]. In concurrence to this, two experimental studies demonstrated an increase in salivary EGF and an increase in ileal EGFR expression and activation just 3 days after massive intestinal resection in rats [143,144].

In the only published human clinical study regarding EGF administration in SBS, the five pediatric patients demonstrated increased carbohydrate absorption and increased overall caloric absorption from enteral feeds [145]. Although salivary EGF plays an important role in intestinal adaptation, it is not the sole discovered saliva-derived mediator of this process. Rather, the adaptive effects of salivary EGF appear to be dependent on concomitant saliva-derived vascular endothelial growth factor (VEGF). In murine models of small bowel resection with sialoadenectomy, complete intestinal adaptation was observed only in animals receiving combined EGF and VEGF supplementation, whereas administration of either factor alone was insufficient [55]. This extraintestinal source of VEGF is further corroborated by studies examining angiogenesis, which found no increase in ileal VEGF expression after intestinal resection, compared to sham surgery in rat models [54].

Insulin-like growth factor-1 (IGF-1) is another molecule of interest. Myofibroblasts from the intestinal crypt mesenchyme are a significant source of IGF-1 [146]. Preclinical experimental studies demonstrated that IGF-1 augments mucosal functional adaptation, primarily through increased activity of sucrase, maltase, and leucine aminopeptidase [147,148]. Major regulators of IGF-1 levels are GLP-2, ghrelin, and PAPP-A2. Ghrelin shows positive effects on structural intestinal adaptation, possibly by stimulating the secretion of both GH and IGF-1 [149]. Expression of PAPP-A2 is also increased by GLP2. PAPP-A2 enhances IGF-1 bioavailability through targeted proteolysis of the IGF binding proteins IGFBP3 and IGFBP5 [150].

### 7.6. Glucagon-like Peptides

Proglucagon is a precursor peptide which is then cleaved in a tissue-specific manner into different active hormones. In the pancreas, proglucagon is cleaved into glucagon and the major proglucagon fragment, while in the intestine, it is cleaved into glucagon-like peptides 1 and 2 (GLP-1 and GLP-2), glicentin, and oxyntomodulin [151]. Experimental evidence consistently shows that the ingestion of nutrients, including macronutrients as well as butyrate, serves as a major driver of GLP secretion [125,152,153]. Higher meal energy content has been shown to elicit greater postprandial secretion of GLP-2 [154,155,156]. In healthy individuals, this negative-feedback mechanism slows gastric emptying and intestinal transit, thereby preventing excessive nutrient delivery to the distal gut [157]. Prahm et al. have demonstrated that high-protein meals lead to highest GLP-2 levels in humans, while high-carbohydrate meals cause the earliest peak in GLP-2 levels [158]. Ingestion of food triggers the depolarization of intestinal L-cells, and secretion of glucagon-like peptides. Sodium glucose co-transporter 1 (SGLT1) is essential for L-cell depolarization and GLP-1 secretion, following oral glucose intake [159,160,161,162]. Multiple studies have demonstrated that nutrient ingestion triggers equimolar release of GLP-1 and GLP-2 into the circulation in physiologic conditions. This reflects their shared biosynthetic pathway and co-secretion mechanism; however, circulating levels may differ due to different post-secretory fates of the two peptides [163,164]. Increased influx of nutrients leads to higher expression of glucagon-like peptides in the remaining bowel. L-cells are most densely populated in the terminal ileum, and in an experimental model of a distal bowel resection with the preservation of the terminal ileum resulted in a significant increase in GLP-1 expression [165]. An experimental rat study has further elucidated the responsible mechanism: presence of fat in the duodenum triggers GLP release through a complex neuroendocrine loop; duodenal enteroendocrine cells release glucose-dependent insulinotropic peptide (GIP), which stimulates the vagal afferent fibers. After passing through the vagal nuclei, signal continues through the efferent signal into the distal small intestine, where it stimulates L-cells to release GLP-1 [166]. GLP-2 secretion is significantly increased after massive small bowel resection, reflected in the significant increase in circulating levels [167,168]. Although GLP-1 expression is increased after massive intestinal resection, there are conflicting reports about post-resection circulating GLP-1 levels. In one study, GLP-1 levels decreased following significant small bowel resection [169], and another study demonstrated a decrease in active GLP-1, despite increased overall GLP-1 levels following RYGB surgery [170]. Another interesting pediatric study demonstrated that PN-dependent SBS patients had lower circulating levels of GLP-1 compared with PN-weaned SBS patients. Additionally, the same study demonstrated a consistent increase in GLP-2 levels, regardless of PN dependence, compared with age- and sex-matched controls [171]. While PN-dependence assumes insufficient enteral nutrition, the presence of oral nutrients in the gut lumen is an adaptive stimulus. Contrary, exclusive parenteral nutrition is deleterious to intestinal adaptation, as evidenced by an early reduction in GLP-2 secretion, which is detectable as early as the third postoperative day in a murine model [101].

Glucagon-like peptide 1 is a peptide hormone with a very short half-life (up to 2 min) secreted by intestinal L cells, which acts through its receptor GLP-1R. GLP-1R is found in various organs: pancreas, intestine, heart, lungs, kidney, as well as the central and peripheral nervous systems. Binding to GLP 1R, GLP-1 increases insulin secretion, reduces glucagon release and reduces the intake of food by centrally suppressing appetite [172]. Both GLP 1 and GLP 2 hormone levels correlate well with satiety [173]. GLP-1’s less addressed effects, like delayed gastric emptying, inhibition of gastric acid secretion, and increased bowel transit time have sparked interest in GLP’s role in SBS. There is also evidence that GLP modulates gut integrity and reduces intestinal permeability [174]. Additionally, experimental animal studies have shown that GLP-1 administration also increased mucosal surface area [125]. All of the above-mentioned effects are beneficial in intestinal adaptation. In a human case series, five patients treated with exendin-4 (a GLP-1R agonist) had slowed gastric emptying with inhibition of the gastric antral motor activity, reduced the number and frequency of bowel movements and were no longer dependent on PN. Additionally, these patients had increased their urine output, suggesting a better hydration status [175]. The design of the study does not allow for the distinction of whether the exhibited positive effect of exendin-4 was a result of increased mucosal surface area or reduced gastrointestinal motility.

Glucagon-like peptide 2 is another peptide hormone with a very short half-life (around 7 min) secreted by intestinal L cells in the distal small intestine and colon [176]. GLP-2 acts by stimulating crypt enterocyte proliferation [177,178,179] and inhibiting intestinal cell apoptosis [180], while reducing gastric acid secretion and gastric peristalsis. GLP-2 also promotes the mucosal repair, increases nutrient absorption, increases mesenteric blood flow, and regulates the intestinal barrier [176,181]. GLP-2 exerts its actions through a highly specific G-protein–coupled receptor (GLP-2R) that signals primarily via cAMP. GLP-2R is not expressed in stem cells; however, it is expressed in the subepithelial myofibroblasts, enteric neurons and enteroendocrine cells in the stomach, small intestine, colon, implying that many of the downstream effects are indirect [151]. GLP-2 stimulates the release of growth factors including IGF-1, EGF, and VEGF from ISEMFs [128,182,183]. An experimental study further elaborates that GLP-2-related activation of enterocyte survival occurs at physiological concentrations, while enterocyte proliferation occurs at high GLP-2 concentrations. Intestinal atrophy that occurs in patients undergoing TPN is likely related to the loss of normal GLP-2 secretion [184]. As reported by Markovic et al., GLP-2 also enhances intestinal epithelial structure through an IGF-1R–dependent mechanism. It increases microvillus length in normal mice, an effect lost when epithelial IGF-1R is absent—indicating that this pathway is required for both baseline microvillus maintenance and GLP-2–induced elongation, possibly through translational regulation of villin synthesis. This response likewise requires intact IGF-1R signaling. These findings show that GLP-2 remodels the brush border primarily by promoting villin-dependent microvillus growth [185]. A study published in 2016 suggests that the antioxidant effect of GLP-2 may be due to upregulation of GRP78 protein, a protein with an important role in oxidative damage prevention [186]. GLP-2 increases intestinal barrier function by inducing enterocyte proliferation and increasing ZO-1 and occludin expression [183]. GLP-2 also enhances intestinal lipid absorption by stimulating nitric oxide (NO)–dependent pathways that promote chylomicron production and facilitate enterocyte lipid handling. This may be further supported by VEGF-mediated signaling and increased mesenteric blood flow [187]. A significant reservoir of intestinal L-cells is located in the colon, and the anatomical SBS type is an important predictor of intestinal adaptation [188]. A human study corroborates this; it has demonstrated that SBS patients without colon and ileum have a blunted postprandial GLP-2 response [189]. Moreover, the trophic effects of GLP-2 administration have not been reproduced when GLP-2 is given alone, even at high doses, suggesting that its full activity requires the presence of luminal trophic factors, particularly nutrients [184,190].

GLP-1 and especially GLP-2 play a critical role in intestinal adaptation. Furthermore, patients with resected ileum, but preserved colon continuity had higher postprandial concentrations of GLP-1 and GLP-2 compared to age and sex matched controls, suggesting an adaptive increase in glucagon-like peptide secretion [191]. Another study in a paediatric population showed that patients with small bowel-colic continuity had higher concentrations of fasting GLP-2, compared with patients with small intestinal end-ostomy [171]. L-cell secretion of GLP-1 and GLP-2 is crucial for intestinal adaptation, and one study of human infants with intestinal dysfunction, associated postprandial GLP-2 concentrations >15 pmol/L with successful weaning from parenteral nutrition, whereas levels below this threshold predicted failure to achieve parenteral independence in 75% of infants by 1 year of age [192]. In addition, baseline circulating GLP-2 levels may serve as a predictive biomarker of therapeutic response. Patients with low PAPP-A2 and low GLP-2 levels at baseline had a higher response rate to teduglutide in one study [150].

Peptide YY (PYY) is secreted by the L-cells in the distal ileum and colon. After being released to the bloodstream, the hormone acts by inducing satiety, reducing gastric secretions, reducing pancreatic exocrine secretions, and reducing gastrointestinal motility. This hormone serves as a pivotal effector of the ileal and colonic brake mechanisms, which attenuate gastric emptying in response to nutrient exposure in the distal ileum and colon [6]. Enterocytes and neurons of the submucosal and myenteric plexus express Y1 and Y2 receptors, which serve as binding sites for PYY [193]. PYY induces satiety by reaching the brain through several different routes, including direct access to areas with a naturally leaky blood–brain barrier, signaling through vagal nerves to the brainstem, and activation of downstream pathways that influence appetite and reward centers. PYY also inhibits gastric acid and pancreatic exocrine secretion primarily by suppressing vagal cholinergic activity and inhibiting enteric neuronal signaling, which together reduce parietal cell activation and pancreatic enzyme release [194,195]. Locally secreted PYY from the L-cells acts in a paracrine fashion, stimulating intestinal crypt cell proliferation, primarily through a Y1R/EGFR/MAPK axis [196]. Experimental administration of peptide YY was shown to enhance both basal and postprandial nutrient absorption in canine models [197].

Short bowel syndrome is also associated with alterations in upper gastrointestinal hormonal signaling, including ghrelin and gastrin. The abrupt removal of distal enteric inhibitory signaling after extensive small bowel resection triggers a temporary hypergastrinemic state associated with gastric acid hypersecretion. However, following adaptation, gastrin concentrations remain elevated in the absence of increased acid secretion [198,199]. Ghrelin levels are higher in patients with short bowel syndrome in both fasting and postprandial state. However, it is not clear whether ghrelin is responsible for the adaptive hyperphagia in the long term [200,201,202].

## 8. Research Gaps and Future Perspectives

Once considered untreatable, short bowel syndrome today has a significantly improved prognosis. The first breakthrough came in 1960s and 1970s when total parenteral nutrition (TPN), and later home parenteral nutrition (HPN), have been recognized as a viable long-term treatment approach [203,204]. However, the long-time use of PN is now known to be a crucial risk factor in the development and progression of intestinal failure associated liver disease (IFALD). IFALD is characterized by inflammation which may be accompanied by cholestasis, steatosis, and fibrosis ultimately leading to cirrhosis [205].

The development of GLP-2 analogues has significantly changed the disease course of SBS patients and allowed for enhanced adaptive response. Teduglutide is a GLP-2 analogue with a long half-life that allows for once daily dosing. In a 24-week placebo-controlled trial, both treatment arms exhibited reductions in parenteral support volume and achieved a decrease of at least one infusion day per week, consistent with ongoing intestinal adaptation in all participants. Patients receiving teduglutide demonstrated a significantly greater treatment effect, with an almost twofold reduction in parenteral support volume compared with placebo [206]. Despite marked improvements in survival and reductions in parenteral nutrition dependence attributable to contemporary management strategies, a significant proportion of SBS patients remain PN dependent. Understanding the mechanisms behind intestinal adaptation is necessary for the development of novel and improvement of current therapeutic strategies. Recognition of the preserved ileum and colon as key reservoirs of enteroendocrine L-cells, together with the identification of hyperphagia as a major component of the bowel adaptive response, has translated into meaningful improvements in clinical outcomes. On the other hand, experimental and clinical evidence remains insufficient to guide strategies aimed at inducing improved bowel structure and function in the setting of limited oral intake, particularly among patients with transient conditions that preclude adequate enteral nutrition. Although this review provides a comprehensive synthesis of the literature on adaptive mechanisms in SBS and its putative regulatory factors, several critical gaps remain. In particular, downstream signaling events mediating the effects of the GLP-2/EGF axis have not been systematically investigated. While Wnt signaling is known to be essential for crypt cell maintenance and proliferative capacity, mechanistic studies directly integrating Wnt activity with GLP-2– or EGF-driven adaptive pathways are currently lacking. Another major gap in current knowledge is the absence of a clearly defined clinical and pathophysiological timeline for functional recovery of the remaining bowel. Future research should aim to delineate whether these constraints to adaptive capacity exist, as it is plausible that intestinal adaptation follows a “use-it-or-lose-it” paradigm at the cellular level. Importantly, maladaptive effects of total parenteral nutrition are evident as early as day three in murine models [101]. Moreover, in the early post-resection period, many patients experience significant comorbidities—such as sepsis, pulmonary embolism, or chronic critical illness—which may impair enteral stimulation and metabolic signaling, potentially resulting in a blunted or delayed adaptive response of the remaining intestine.

Promoting intestinal adaptation represents a key frontier in the treatment of SBS patients. Further mechanistic insight into downstream effectors of key regulatory pathways will unveil additional treatment targets, enabling a shorter period of PN dependence and lower long-term mortality. Future prospective studies will be essential to validate mechanism-based therapeutic strategies and translate these insights into improved clinical outcomes.

## Figures and Tables

**Table 1:** An overview of hormones and their implicated roles in short bowel syndrome. Glucagon-like peptides 1 and 2, as well as peptide YY are secreted by the L-cells, which are most abundant in the distal ileum and colon. GLP-2 exerts its effects on crypt cells indirectly through downstream effectors like EGF, VEGF, IGF-1 which are secreted by subepithelial myofibroblasts. Further downstream pathways have not been elucidated in SBS. The exception to this is the mechanism of apoptosis inhibition, which is known to include upregulation of Bcl-w and downregulation of p38 MAPK and Bax proteins in enterocytes.

Hormone	Implicated Effect in Short Bowel Syndrome
GH	Increased small bowel absorptive capacity Increased intestinal adaptation
EGF VEGF	Increased ISC mitosis Decreased enterocyte apoptosis Reduced gut permeability Angiogenesis
GLP-1	Delayed gastric emptying Suppression of gastric acid secretion Increased bowel transit time Reduced gut permeability
GLP-2	Increased ISC proliferation (pharmacologic concentrations) Decreased ISC apoptosis (physiologic concentrations) Suppression of gastric acid secretion Increased mesenteric blood flow Increased villin dependent microvillus growth Reduced gut permeability
PYY	Ileal and colonic brake
Aldosterone	Fluid and sodium conservation
Ghrelin	Adaptive hyperphagia
Gastrin	Gastric hypersecretion

## Data Availability

No new data were created or analyzed in this study. Data sharing is not applicable.
